# Design Principles Of Inorganic‐Protein Hybrid Materials for Biomedicine

**DOI:** 10.1002/EXP.20240182

**Published:** 2025-03-06

**Authors:** Hao Liu, Xiaohui Liu, Hui Jiang, Xuemei Wang

**Affiliations:** ^1^ State Key Laboratory of Bioelectronics (Chien‐Shiung Wu Lab) School of Biological Science and Medical Engineering Southeast University Nanjing Jiangsu China

**Keywords:** biosynthesis, interventional therapy, living materials, protein coronas, smart materials

## Abstract

Inorganic protein hybrid materials (IPHMs) due to editable structure present unrivalled potential at the intersection of synthetic biology and materials science. The synthesis of IPHMs with a high degree of biosafety from bioactive units represents a shift in material design and synthesis. This paper focuses on a review of the structural basis and design principles of proteins for the synthesis of IPHMs with specific physical and chemical functions. It also provides a valuable reference for the design of emerging IPHMs through the conformational relationship of the IPHMs, which extends the potential applications of IPHMs. In addition, the construction strategy of the reaction system for the synthesis of hybrid materials is analyzed from the perspective of synthetic biology. The possibility of engineering and batch synthesis of IPHMs is discussed. Based on the physicochemical properties of different hybrid materials and the applied‐oriented research on biomedical optical imaging and multimodal therapy, the idea of synthesis of in situ hybrid materials is proposed. Ultimately, the trends and challenges of synthetic biology for IPHMs are speculated in detail.

## Graphical abstract

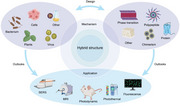

## 1 Introduction

The design and synthesis of nanomaterials with specific scales, structures, and functions for different medical scenarios is a development trend, which could promote the clinical transformation of nanomaterials [1, 2]. From a scale perspective, smaller‐sized nanomaterials are easily enriched at the tumor site and can be metabolized by the kidneys [3, 4]. From the perspective of morphology and structure, nanosheets have a larger specific surface area, which gives them a higher loading rate for drugs [5, 6, 7]. To achieve a medical optical diagnosis, fluorescent (FL) quantum dots, nuclear magnetic contrast (MRI) agents, surface‐enhanced Raman scattering (SERS) probes, etc. are required [8, 9]. In addition, nanomaterials with photothermal, photodynamic, or chemodynamic effects are required for physicochemical‐related therapies for diseases [10, 11]. The method of designing and synthesizing nanomaterials with specific functions while meeting the premise of biosafety has already given us the answer in nature [12, 13]. Having evolved through nature, it provides us with a range of post‐processed novel designs and high‐performance structures at the nanoscale. It is composed of proteins and inorganic nanostructures, which are inorganic protein hybrid materials (IPHMs).

The surface of the IPHMs is composed of proteins, while the core is an inorganic mineral structure. The benefit of such a structure is better biosafety. The surface proteins do not affect the movement and adsorption of biomolecules in the bloodstream [14, 15]. It can effectively protect the integrity of the core inorganic structure in subcellular structures with extreme microenvironments such as lysosomes, so that they can continue to function [16, 17]. The targeted regulation of inorganic material formation by proteins in living organisms is achieved through processes such as nucleation, growth, phase transition, and self‐assembly [18, 19]. Alternatively, inorganic nanomaterials form protein coronas after adsorbing proteins through electrostatic or other non‐covalent interactions [20, 21]. The IPHMs based on protein growth from bottom to top are usually influenced by factors such as protein sequence and secondary structure, and the tertiary structure formed by different inorganic ions is also different.[22, 23] The formation of fibrous peptide structures provides sites for crystal nucleation [24, 25, 26]. Proteins rich in thiol groups are more likely to synthesize gold nanoparticles, while peptide segments rich in isoleucine can regulate crystal growth [27, 28]. Moreover, chiral amino acids have different binding energies with different crystal planes, which can regulate the directional growth of crystals to obtain chiral nanostructures [29, 30]. The different types of substances synthesized result in different properties. Iron oxide nanoparticles exhibit magnetic and chemical kinetic effects, gold nanoparticles exhibit plasmon resonance and photothermal effects, and rare earth quantum dots exhibit fluorescence and photodynamic effects [31, 32, 33].

In synthetic biology, the synthesis of IPHMs from bioactive units is a dynamic and controllable process that provides a designable source [34, 35, 36]. This is not like in vitro chemical synthesis. The advantages of biosynthesis are sustainability, low pollution, batch production, and higher biological safety of the synthesized IPHMs [37, 38, 39, 40]. Due to the presence of ferritin, magnetotactic bacteria can synthesize magnetosomes, diatoms deposit silica in intracellular vesicles, and extracellular matrix proteins in tumor tissues provide the basis for nucleation of microcalcifications [41, 42, 43]. These are the biological foundations of IPHMs. In addition, the occurrence of some pathological events can also generate IPHMs, such as metal nanoparticles produced during the treatment of tumors with metal complexes, excessive calcium ions forming stones, mineralized nodules in the kidneys, and so on [44, 45, 46, 47]. The IPHMs can be guided to generate specific biological functions for disease diagnosis or treatment through controllable synthesis and design in advance. For example, the surface of tumor cells undergoes silicification or calcification, becoming a tumor whole‐cell vaccine for immunotherapy of tumors [48, 49]. Engineering *E. coli* is used to synthesize immunogenic gold nanoparticles in batches in fermentation tanks [50, 51]. Alternatively, gold nanoparticles can be generated within cells to achieve in situ SERS mapping of tumors.[52, 53]. These are creative synthesis strategies and methods.

**SCHEME 1:**
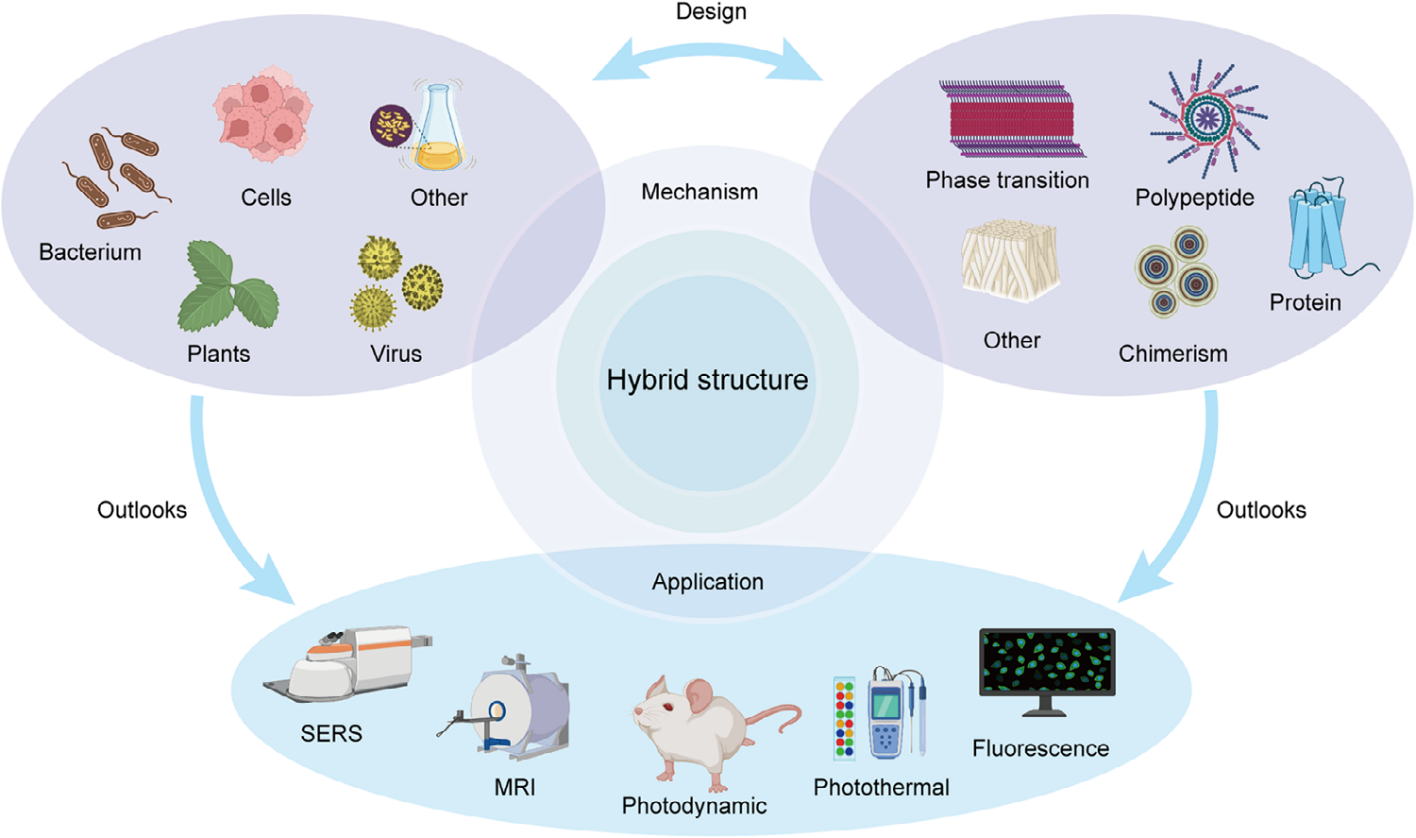
Schematic diagram of hybrid material design and biological applications.

In this review, a detailed summary and critical analysis were conducted from the perspective of the design principles and synthesis laws of IPHMs (Scheme 1). The relationship between the selection and function of protein sequence, structure, peptides, and inorganic ions at the molecular level is summarized. The strategies for constructing hybrid materials in synthetic biology have been summarized, such as cells, bacteria, plants, viruses, etc. On this basis, the potential applications of hybrid materials with different functionalities in biomedical fields have been summarized. And we also put forward our views and speculations on the future development of this field.

## 2 Protein Foundation of IPHMs

### 2.1 IPHMs Based on Native Proteins

**FIGURE 1:**
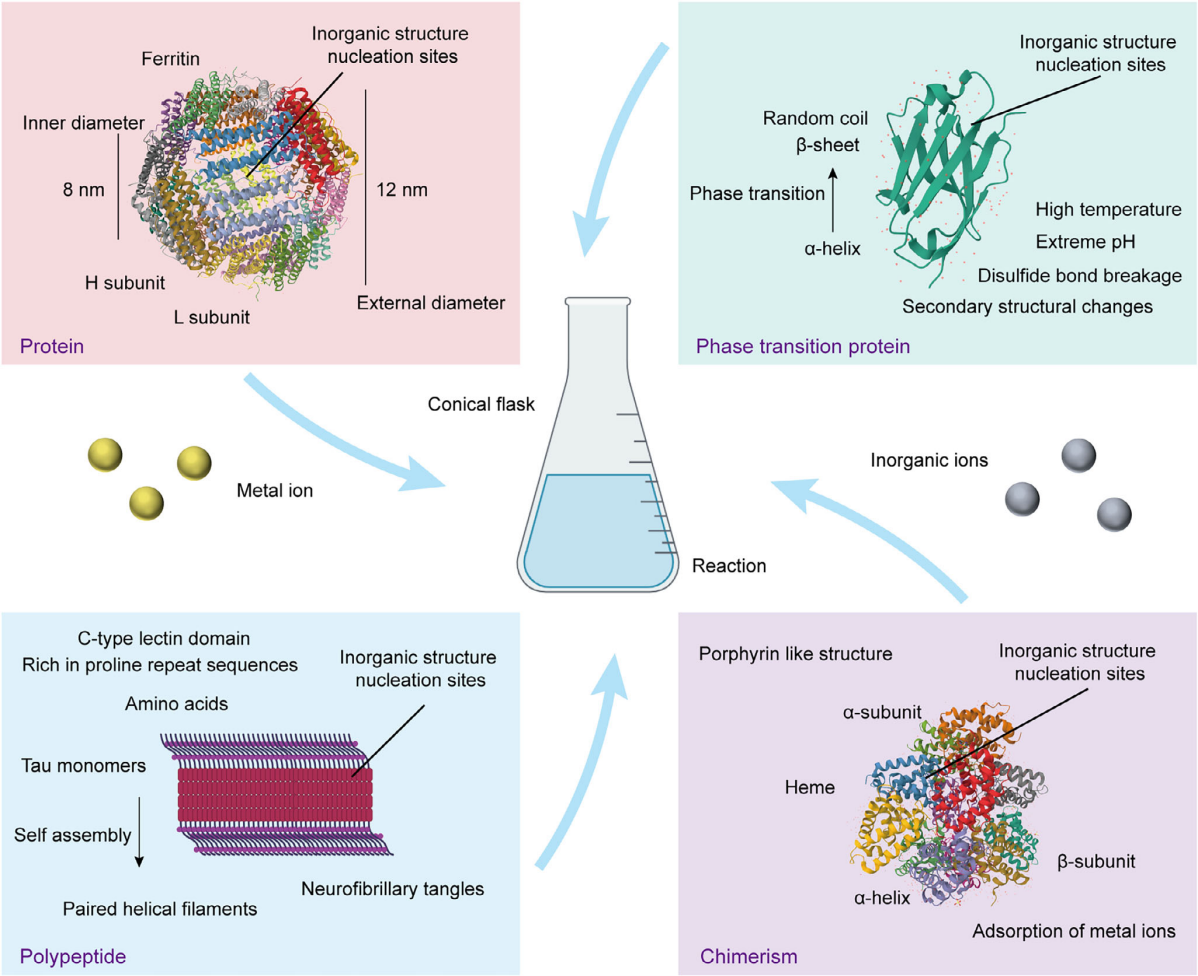
Schematic illustration of the design principle of proteins. Describe from the perspectives of proteins, phase transition proteins, peptides, and chimeras.

**FIGURE 2:**
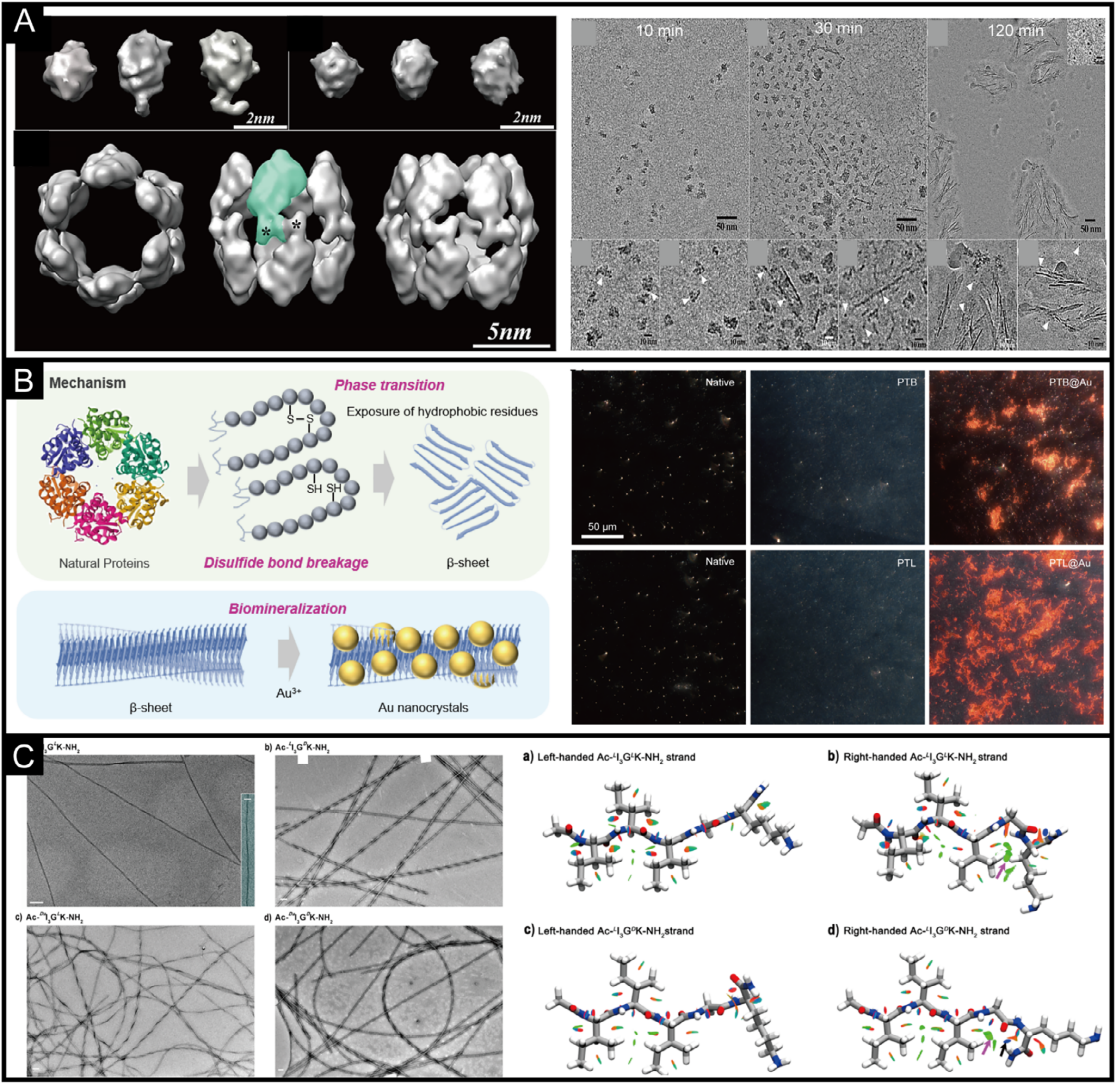
(A) Reconstruction model and cryo‐electron microscopy (Cryo‐EM) image of calcium phosphate (ACP) mineralization with full‐length amelogenin rM179 [76]. Copyright 2011, U.S. National Academy of Sciences. (B) A structural model for phase transition proteins and dark field images of mineralized gold nanocrystals [113]. Copyright 2024, Elsevier. (C) Negative‐staining TEM imaging of self‐assembled peptide nanofibrils and its reduced density gradient (RDG) gradient isosurfaces for single β‐strands [128]. Copyright 2023, American Chemical Society.

The active molecules of proteins are the basis for the formation of IPHMs, and the design of proteins from natural proteins to phase transition proteins, peptides to amino acids is crucial (Figure 1 and Table 1). The tertiary structure of native proteins can provide growth space and nucleation sites for IPHMs [54, 55, 56]. For example, hydroxyapatite based on protein formation, magnetite, calcium carbonate, manganese oxide, silica, and so on, all belong to IPHMs [57, 58, 59, 60]. Collagen fibers are assembled from collagen molecules, with a regular arrangement and certain directionality [61, 62]. There are gaps between different fibers, with a width of approximately 0.6 times that of collagen fibers [63, 64]. The space formed is just enough to accommodate the growth of hydroxyapatite crystals to form IPHMs. The tertiary structure composed of enamel protein and collagen fibers is similar, which can inhibit the lateral growth of crystals, resulting in the formation of hydroxyapatite as a long strip structure along the c‐end [41, 65, 66]. MBP‐MamC loop structure (Mms13) is an anchoring protein that regulates the crystallization of magnetosomes, and its possible mechanism is the binding with iron oxide crystals [67, 68, 69]. The presence of a single nucleation site, rather than multiple, results in the formation of a single crystal of magnetosomes [58, 70, 71]. Twins occasionally appear in the direction of magnetosomes (111), but these twins do not affect the magnetism of magnetosomes because the direction of the crystal plane is easily magnetized [72, 73]. The crystal morphology of magnetosomes formed under different conditions varies, including octahedron, cube, dodecahedron, and morphology formed by twisted extension [71, 74, 75]. Ferritin nanocages are assembled from active H‐type subunits and inactive L‐type subunits, with a hollow structure in the middle (Figure 2A). The nucleation sites of iron crystals are two, and their structure and sequence are highly conserved [76]. These can all serve as protein templates required for the synthesis of IPHMs. The formation of this crystal shows a positive correlation with reaction time.

**TABLE 1:** The different types of protein basis of IPHMs.

Synthesis method	Organic structures	Inorganic structure
Chemical synthesis	Native proteins	Crystal/Complex
	Phase transition proteins	Crystal/Complex
	Peptides and amino acids	Crystal/Complex
Biosynthesis	Bacterium	Crystal/Complex
	Cells	Crystal/Complex
	Plants	Crystal/Complex
	Virus	Crystal/Complex

Another part is not only related to the tertiary structure of proteins but also to the catalytic or oxidative efficiency of proteases [77, 78]. Multicopper oxidase (MCO) and manganese peroxidase (MNP) can oxidize manganese to form manganese oxide nanoparticles [26, 79]. The inorganic structure of IPHMs formed by enzyme catalysis is mostly amorphous, occasionally producing crystalline structures, but it usually with poor crystallinity [80, 81]. The fundamental reason is the embedding of protein molecules, which can carry a large amount of protein while also reducing the physical and chemical properties of inorganic structures. In addition, the phenomenon of non enzyme‐catalyzed deposition also exists [82, 83]. There is an S‐layer structure containing proteins on the surface of bacteria. This structure is prone to detachment, has strong adhesion, carries charges, and can highly self‐assemble [84, 85]. This can adsorb free metal ions and form an IPHM coating on its surface. This coating will become saturated to a certain extent and cannot deposit indefinitely.

Calcium carbonate has six different crystal and amorphous structures, including aragonite, calcite, monohydrate calcium carbonate, aragonite, hexahydrate calcium carbonate, and non‐static calcium carbonate [86, 87, 88]. The protein factors that affect the structure of calcium carbonate can be divided into insoluble matrix proteins and soluble matrix proteins, which play different roles in the formation of crystals [89, 90, 91]. For example, it provides nucleation sites, controlling orientation, size, etc. A class of soluble proteins rich in acidic amino acids can inhibit the formation of crystals by embedding them in the crystals [92, 93]. Insoluble proteins provide more nucleation sites and regulate crystal growth. Silicone protein is a protease that regulates the deposition of silica. Mature silica proteins assemble into fine filamentous structures, and silica begins to deposit from both ends [94, 95]. The maturation and assembly of silicon proteins are related to their phosphorylation.

The formation factors of IPHMs based on natural proteins are diverse. Firstly, it is necessary to consider selecting and designing proteins with tertiary structures and spaces for nucleation sites and crystal growth. Secondly, it is necessary to consider the type of oxidase and oxidation efficiency, which can promote the deposition of IPHMs. Thirdly, the solubility and acid‐base properties of proteins also need to be considered. Fourthly, the influence of protein phosphorylation sites should be considered. Fifthly, if non enzymatic factors are considered, attention should be paid to issues such as protein charge, hydrophilicity, and hydrophobicity. New substances can be obtained by replacing the original metal ions. It is very important to imitate natural proteins during design, which is half the success.

### 2.2 IPHMs Based on Phase Transition Proteins

The understanding of phase transition proteins is that natural proteins are influenced by external factors, leading to changes in the tertiary and secondary structures of proteins. This includes temperature, pH of buffer solution, ion concentration, etc. [96, 97, 98]. A typical example is that higher ambient temperatures can alter the conformation of bovine serum albumin (BSA), which is the transformation of the α‐helix into a β‐sheet (Figure 1). The protein structure rich in β‐sheets can provide nucleation sites for inorganic ions, inducing the formation of IPHMs. It's like amyloid protein. The reason why the ion concentration and acid‐base properties in buffer solutions affect proteins is that the isoelectric points of different proteins are different [99, 100]. The pH value under physiological conditions is 7.6, and proteins with high isoelectric points have a greater degree of proton loss in the amino binding solution of the alkaline amino acid side chains than acidic amino acids [101, 102]. At this time, proteins undergo a certain degree of phase transition, making it easier for them to adsorb inorganic ions and produce IPHMs [103, 104]. Under extreme temperature and acid‐base conditions, proteins undergo more structural damage than regular phase transitions. The ultimate result is enhanced hydrophobicity of the protein surface, enhanced interactions between polymers, and reduced conformational stability of the protein, which precisely meet the conditions for the deposition of inorganic structures [105, 106]. This is why some pathological microenvironments are prone to the formation of stones. Optical factors can also achieve the phenomenon of protein aggregation. Ultraviolet radiation can oxidize methionine in proteins to methionine sulfoxide, which affects the interactions between components through electrostatic interactions, resulting in the formation of non‐fibrous oligomers [107, 108]. This type of IPHMs usually difficult to control the formation of inorganic structures, and proteins have no biological activity. However, the advantage is that the selection of protein templates is universal and less affected by secondary structures.

A more controllable way is to achieve protein phase transition by cutting off disulfide bonds in the protein [109, 110]. The secondary structure of the phase transition protein obtained by this method is more controllable, and its conformation can be controlled by the concentration of salt ions in the solvent environment [111, 112]. This phase transition protein can adhere to solid interfaces, forming hydrophobic and fibrous structures [113]. It can nucleate and crystallize inorganic ions. For example, gold ions can form fibrous IPHMs at the phase transition protein interface of BSA (Figure 2B). Based on the optical scattering principle of gold nanocrystals, a dark field microscopic imaging structure diagram can be obtained. If gold ions are replaced with simulated body fluid buffer (SBF), hydroxyapatite crystals will form at the interface formed by phase transition proteins [114, 115]. That is to say, different types and structures of IPHMs can be constructed by replacing different inorganic ions. Another factor to consider is the selection of reagents used to cleave disulfide bonds, such as tris(2‐carboxyethyl)phosphine (TCEP), dithiothreitol (DTT), β‐mercaptoethanol (BME), glutathione (GSH), and dithioerythritol (DTE) [110]. The properties of different disulfide bond reducing agents are different and may affect the crystallization of inorganic ions. TCEP can simultaneously act as a chelating agent that requires metal ions, which is competitive with phase transition proteins. BME can preserve the biological activity of proteins to the maximum extent, while DTT can maintain the state of disulfide bond cleavage. It is necessary to select appropriate disulfide bond reducing agents based on the structure and functional requirements of the target IPHMs.

### 2.3 IPHMs Based on Peptides and Amino Acids

Amino acids and peptides are the most fundamental units in protein composition, and they are also crucial in the construction of IPHMs. Small fragments of acidic peptides are added to the reaction system of calcium carbonate crystallization, which can form calcite containing (110) and (012) crystal faces [116, 117]. This acidic peptide can be a repeat sequence of phenylalanine (Phe) and aspartic acid (Asp). In the central region of the layered structure of pearls, acidic peptides are embedded within it, suggesting that acidic peptides may provide nucleation sites for infinite structures [118, 119]. This acidic peptide embedded inside the crystal is an endoplasmic protein that can cause changes in the physical parameters and anisotropy of the crystal, such as coherence length and diffraction angle spread [116, 120]. If chiral Asp is added separately to the crystallization system of calcium carbonate, the morphology characteristics of the formed crystals will show the chiral characteristics of amino acid molecules [2, 121]. Cysteine (Cys) is used for the formation of gold nanocrystals due to the presence of sulfur, as it forms gold sulfur bonds [29, 30]. When chiral Cys is used to regulate the growth of chiral gold nanocrystals, chiral elements form triple symmetry and quadruple symmetry in the (111) and (100) directions, respectively. The morphology of IPHMs synthesized from chiral Cys is cubic with a side length of approximately 150 nm [30]. The vertices protrude, and the edges connecting the two vertices are divided into two.

The three‐dimensional structure constructed by peptides can simulate the tertiary structure of natural proteins, providing binding sites for inorganic ions. Similar to the nanospheres formed by amelogenins, there are dense hydrophobic cores inside and hydrophilic fragments connected externally [122, 123]. The basis for this self‐assembly is the A and B domains, which can undergo structural changes under the control of acidity, alkalinity, and ion concentration [124, 125]. This change is an important factor affecting the formation of inorganic nanoparticles. In addition to biomimetic structural peptides, β‐sheets in secondary structures have also been found to form nanofibers [126, 127]. This type of nanofiber can provide sites for the formation of IPHMs. It's like amyloid protein fibers (Figure 2C). Peptides that form amyloid‐like structures are usually selected to act on the structural units formed by fibers, which are KLVFFAECG [24, 128, 129]. Alternatively, convertible peptides can be designed to form rotating fibers, similar to collagen fibers. Such as C_6_RVRRF_4_KY, Ac‐KI_4_K‐NH_2_, ^L^I_3_^L^K, and so on [106, 130, 131, 132]. Different inorganic ions incubating fibrous peptides can obtain fibrous IPHMs of different substances, but the drawback of this design is that structural fixation can only form fibrous structures.

Among the 20 amino acids, different acidity and alkalinity can also lead to different affinities and binding forces towards the same inorganic ions. The binding force between histidine and zinc ions is the strongest [133]. The possible reasons for the binding of amino acids with multiple metal ions are the electrostatic interactions of side chains, redox reactions, and chelation of metal ions [134, 135]. IPHMs can be synthesized using a single type of amino acid, short peptides can be constructed or IPHMs can be chosen.

The design of IPHMs starting from peptides requires more consideration of the structure that peptides can ultimately form and the selection of inorganic ions. It is usually possible to mimic the structure of natural proteins from a biomimetic perspective or extract peptide fragments from natural proteins that have the function of guiding inorganic ion biomineralization or deposition. Then, the adsorption or affinity of amino acids for different inorganic ions can be utilized to screen for different repetitive short peptides or IPHMs of different substances.

### 2.4 Metal Ion Complexes

Proteins or protein aggregates embedded with inorganic ions also belong to IPHMs. Hemoglobin containing iron ions participates in oxygen transport, plasmic blue bodies containing copper ions participate in electron transfer, carboxypeptidases containing metal ions have catalytic activity, and so on [136, 137, 138, 139]. It is possible to achieve the desired function by imitating it. In addition, inorganic nanoparticles containing protein crowns are relatively easy to adsorb metal ions to form single‐atom nanoenzymes, thereby obtaining catalytic activity [140, 141]. However, the single‐atom nanoenzyme formed by this method has poor stability due to the chaotic structure of the protein, and the uncertainty of the structure leads to difficulties in its design.

## 3 IPHMs of Intracellular Biomineralization

### 3.1 Bacterium

**FIGURE 3:**
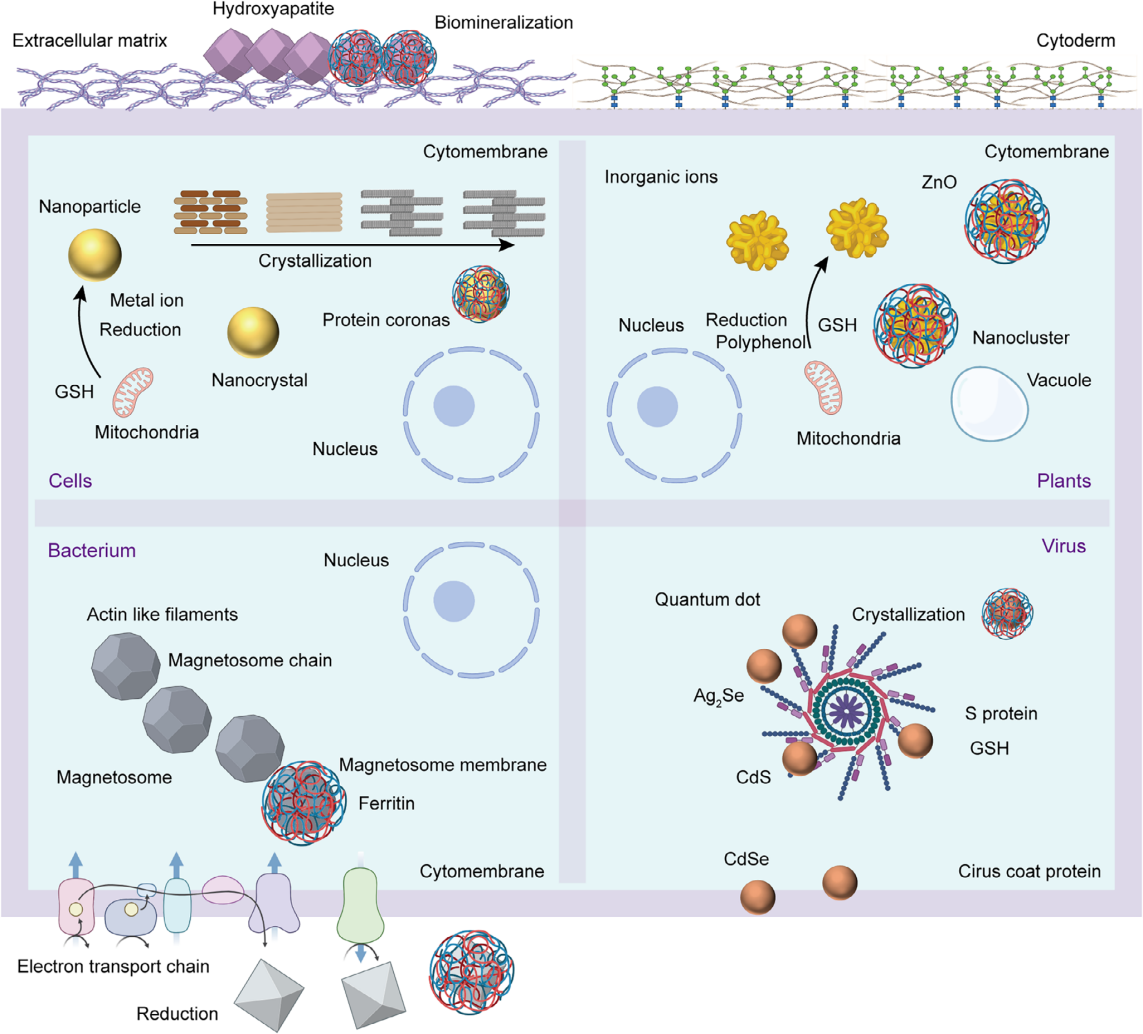
Schematic diagram of biosynthesis of IPHMs. The carriers of biosynthesis are mammalian cells, bacteria, plants, and viruses.

**FIGURE 4:**
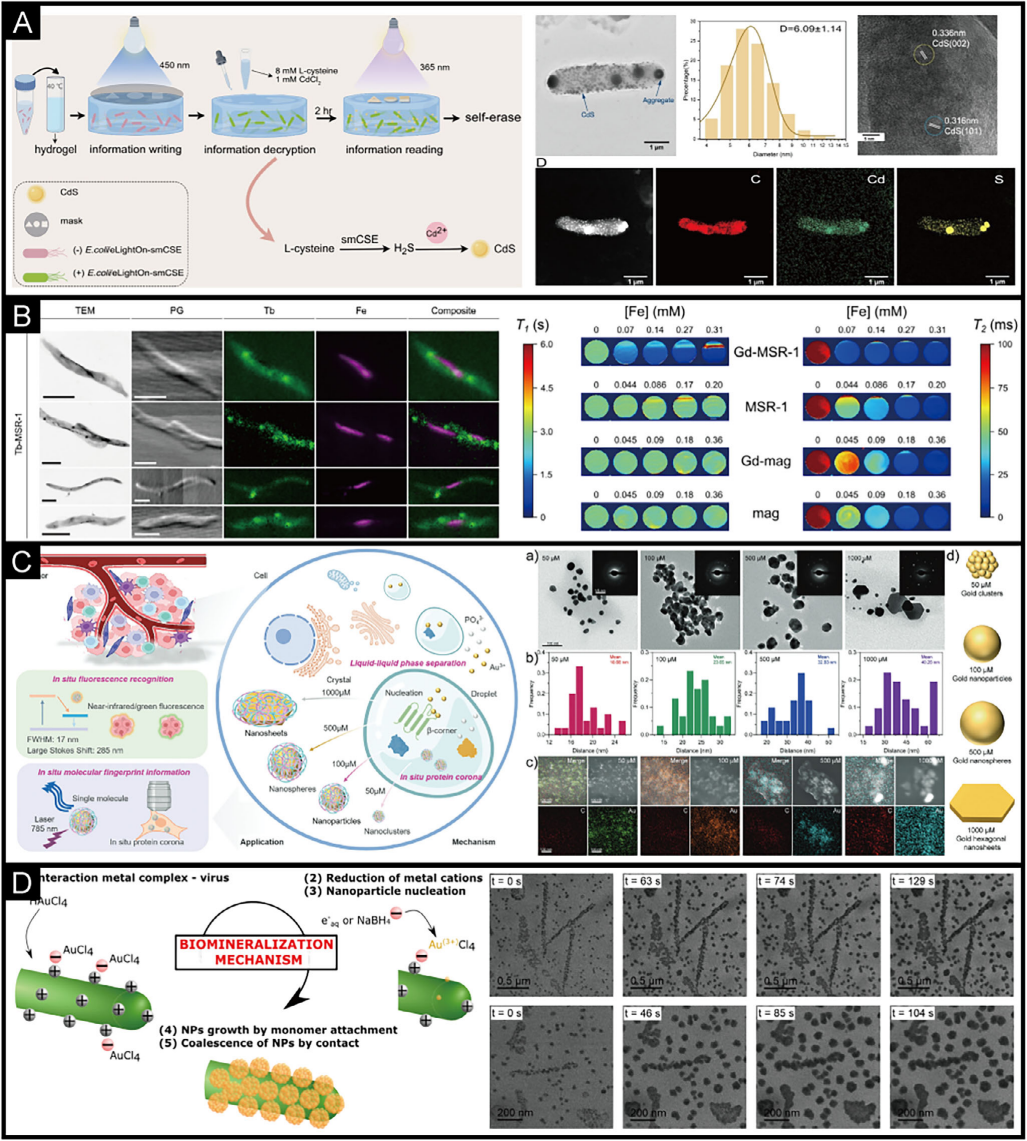
(A) The process of in situ synthesis of cadmium sulfide (CdS) hybrid quantum dots in *E. coli* and transmission electron microscopy (TEM) of *E. coli* after biomineralization [50]. Copyright 2024, Wiley‐VCH. (B) TEM, phase‐gradient (PG), and scanning X‐ray fluorescence microscopy (SXFM) images of Tb‐MSR‐1 and T1 (left) and T2 (right) parametric maps [158]. Copyright 2023, Elsevier Ltd. (C) A schematic diagram of the biomineralization of gold nanocrystals with protein coronas in tumor cells and the morphological and structural characteristics of gold nanocrystals [52]. Copyright 2023, Wiley‐VCH. (D) Schematic diagram of the formation of gold hybrid nanoparticles on tobacco mosaic virus (TMV) and TEM of the relationship between time and synthesis kinetics [200]. Copyright 2023, American Chemical Society.

The synthesis of IPHMs in active units is an innovative method, which includes bacteria, cells, plants, and viruses (Figure 3 and Table 1). Some scholars refer to it as living materials, but from the perspective of matter and materials science, it is an IPHMs. The important foundation of synthetic biology is bacteria, while the foundation of nanomaterial biological applications is safety [142, 143]. The nanomaterials synthesized by bacteria are IPHMs with protein coronas or membrane like structures on the surface, which can meet the requirements of biological safety while being synthesized in bulk. The deposition of manganese nanostructures on the S‐layer structure of bacterial surfaces is a non enzymatic catalytic process, which is related to the structure of the bacterial surface [144, 145]. When screening bacteria for manganese deposition, factors such as surface adhesion, complexity of the extracellular matrix, and the presence of charges need to be considered [146, 147]. This intense oxidation deposition process will not affect bacterial activity, which can consider whether the outer matrix layer is prone to detachment. It is worth noting that, intermediate valence state products or end products with catalytic activity will be produced during the deposition of metal ions. This can promote the deposition of nanostructures through this self‐catalytic process, such as iron and manganese [146, 148, 149]. Autocatalysis can also be achieved by doping different metal ions in the culture medium, and soluble elements such as manganese, iron, copper, and cobalt are usually selected [50, 150, 151, 152]. Rare earth quantum dots can also be synthesized on the surface of bacteria through non enzymatic processes, and the synthesis of cadmium sulfide requires the introduction of hydrogen sulfide gas to supplement sulfur elements (Figure 4A). Cadmium sulfide quantum dots exhibit an aggregated state on the surface of bacteria, which is not uniformly distributed [50]. This indicates that the controllability of this non enzymatic synthesis of IPHMs is not high.

A typical case of gene and protein structure regulation in bacterial biosynthesis of IPHMs is the synthesis of magnetosomes by magnetotactic bacteria [72, 153]. It was found that membrane protein (FezA) and ATPase transporter protein (FezB) can regulate iron excess and deficiency in *C. difficile*, which are necessary for the formation of magnetic bodies [68, 154]. Unlike non enzymatic processes, the generated magnetosomes are evenly distributed in bacteria and do not aggregate irregularly. Magnetosomes mostly exist near the cell membrane and are enveloped by vesicles [155]. Magnetotactic bacteria are a type of bacteria that have spiral, spherical, rod‐shaped, arc‐shaped, and other forms. For example, bilophococcus and aquaspirillum. The morphology of magnetosomes produced by spiral magnetotactic bacteria is cubic octahedron, spherical magnetotactic bacteria produce prismatic magnetosomes, arc‐shaped magnetotactic bacteria produce bullet shaped magnetosomes, and other irregular shapes [122, 156]. When other metal ions are enriched in the microenvironment of magnetotactic bacteria synthesizing magnetosomes (Figure 4B), they are also doped in crystalline magnetosomes [157, 158]. When elements similar to the valence state of iron replace iron, new substances are produced. The design and synthesis of this type of IPHMs can achieve selectivity for specific functions by screening bacterial species and regulating the microenvironment of bacterial mineralization, and gene expression levels.

The key to the mass and commercial production of IPHMs lies in bacterial screening, bacterial construction, production, and the separation and purification of IPHMs. When designing and synthesizing IPHMs, the reuse of by‐products, such as inorganic ions in intermediate valence states, may need to be considered.

### 3.2 Cells

The production of IPHMs in mammalian cells is divided into two types based on the mechanism of mineralization: physiological and pathological IPHMs [159, 160, 161]. Ameloblasts and odontoblasts interact to synthesize hydroxyapatite to form teeth, while osteoblasts and osteoclasts produce bone or cartilage tissue [90]. These are physiological processes. Structural proteins can directly form IPHMs with inorganic ions, while regulatory proteins regulate the spatiotemporal expression of structural proteins through signaling pathways to control the formation of IPHMs. Typical structural proteins include amelogenin, amelogenin, and collagen, while regulatory proteins include runt‐related transcription factor 2 (RUNX2), bone morphogenetic proteins (BMP4), and muscle segment homeobox (MSX2) among others [88, 162]. In the process of crystal nucleation and growth controlled by structural proteins, proteases degrade structural proteins under specific spatiotemporal conditions to control the direction of crystal growth. For example, matrix metalloproteinase 20 (MMP‐20), kallikrein‐related peptidase (KLK4), etc. [163, 164]. The mechanical properties of physiologically mineralized IPHMs are excellent, as the complex and controllable formation process leads to higher crystallinity. The design of IPHMs for this physiological mineralization typically requires an understanding of the biological mechanisms of mineralization.

The IPHMs formed by pathological mineralization are relatively uncontrollable, similar to the process of bacteria depositing IPHMs through non enzymatic reactions. When the metabolism of inorganic ions is disrupted, resulting in excessive inorganic ions in the microenvironment, IPHMs are deposited on the protein of the fibrous structure. Similar to kidney stones [165, 166, 167]. The microenvironment of inflammatory and traumatic sites is usually acidic, and there is aggregation of proteins such as platelets, which can also lead to the production of IPHMs [42, 168, 169, 170]. The effect of IPHMs on the body is negative. An example of this type of IPHMs is calcium carbonate produced at atherosclerotic sites.

In addition, the pathological microenvironment can synthesize IPHMs, indicating whether in situ synthesis of IPHMs with therapeutic or diagnostic effects can achieve diagnosis and treatment of the lesion site [171, 172]. The tumor microenvironment is acidic and rich in extracellular matrix proteins, which can lead to microcalcification. It was found that the appearance of microcalcifications prolongs the survival period of tumor patients, which can effectively control tumor proliferation [173, 174, 175]. Metal drugs are chosen to treat tumors by synthesizing metal‐based IPHMs at the tumor site, such as fluorescent gold nanoclusters, platinum nanoparticles, etc. [176, 177, 178]. Gold ions can be biomineralized within tumor cells to produce gold nanoparticles with protein coronas of different sizes and morphologies, which are related to the concentration of gold ions [52, 179]. The gold nanostructures formed by low to high concentrations of gold ions at the tumor site include gold nanoclusters, gold nanoparticles, gold nanospheres, and gold nanosheets (Figure 4C). Gold nanoclusters exhibit fluorescence effects, gold nanoparticles exhibit surface plasmon resonance effects, and gold nanosheets have a larger specific surface area. Platinum drugs can produce platinum nanoparticles in the blood and tumor tissue, which can stimulate the immune system [180]. The biosynthesis of IPHMs at the lesion site is limited by the microenvironment of synthesis, which is not easily altered by external factors. Therefore, the design of IPHMs requires a reliable introduction of precursor chemical properties, concentration, and structure.

### 3.3 Plants

Compared to bacteria and mammalian cells, plants play a different role in natural evolution, which is carbon sequestration. Algal biomineralization of inorganic silicon is quite common [181, 182]. Different types of diatoms produce different structures of silica, and specific morphologies of silica can be synthesized by screening the types of diatoms [183, 184]. It should be noted that the protein isolated from silica is a highly anionic phosphoprotein called silaffin‐2. After mixing silaffin‐2 and organic matrix in vitro, soaking silicon elements can generate porous silica [134, 185]. Gene editing can be used to modify the expression of silaffin‐2 in diatoms to promote the formation of silica. From the microstructure of diatoms, the cell wall is a key factor in the synthesis of silica. Excessive or insufficient concentration of intracellular soluble silicon elements is not conducive to the deposition of silica on the cell wall [183]. The cultivation environment should control the silicon content.

The most abundant polyphenolic substances in plants besides cellulose are gallic acid, catechins, quercetin, tannic acid, and arbutin, among others. Polyphenolic substances have strong reducing properties and can reduce metal elements in soil to obtain inorganic nanostructures [186, 187, 188, 189]. Coordination bonds will be formed between phenolic hydroxyl groups and metal ions to stabilize the structure of nanoparticles [190, 191]. These polyphenol coordinated metal nanoparticles carry negative charges and are prone to adsorbing proteins, resulting in the formation of IPHMs. Due to the dominant reducing nature of polyphenols in plants, proteins are modified by electrostatic adsorption on the surface of nanoparticles during the formation of IPHMs [192, 193]. In addition, electron transfer occurs in plant endocysts during photosynthesis converting gold (III) to gold (0) to obtain gold nanoparticles [194]. When designing plans for plant synthesis of IPHMs, polyphenol reduction and photosynthesis can be utilized.

### 3.4 Virus

Viruses are well‐structured nanoparticles of 30 to 200 nm, consisting of an outer layer of structural proteins [195, 196]. The interior is nucleic acids. Viruses come in a variety of forms including helical, icosahedral, fibrous, and complex [197, 198]. The structure of proteins with easily modifiable surfaces has similarities to that of induced biomineralization, which is often used as a protein template for IPHMs. The tobacco mosaic virus is a tubular virus with a length of 300 nm and a diameter of 18 nm [196, 199]. The surface protein is structurally stable, and it can maintain its structure at high temperatures and extreme acid‐base conditions [200]. The surface proteins are positively charged, which enables nucleation and crystallization of gold nanoparticles (Figure 4D). It presents a fibrous morphology similar to the tobacco mosaic virus [200]. Interestingly, the formed gold nanoparticles exhibit a monodisperse state, which is the advantage of virus synthesis of IPHMs [201, 202, 203, 204, 205]. The focus of virus synthesis of IPHMs is on surface proteins. Firstly, the chemical groups with metal binding ability can promote the deposition of inorganic elements, so the synthesis of IPHMs can be achieved by modifying surface proteins. Secondly, surface charge is related, and negative charges are more likely to adsorb inorganic elements to form IPHMs. Thirdly, the morphology of IPHMs is related to the morphology of the virus, and different forms of viruses can be replaced. Finally, it is related to the synthesis microenvironment such as salt ion concentration and acid‐base properties. Of course, different viruses have different affinities for different inorganic elements, which requires subsequent screening. And there will be relatively more research on the synthesis of fluorescent quantum dots by viruses.

## 4 Biological System Improvement

### 4.1 Disease Therapy Based on IPHMs

**FIGURE 5:**
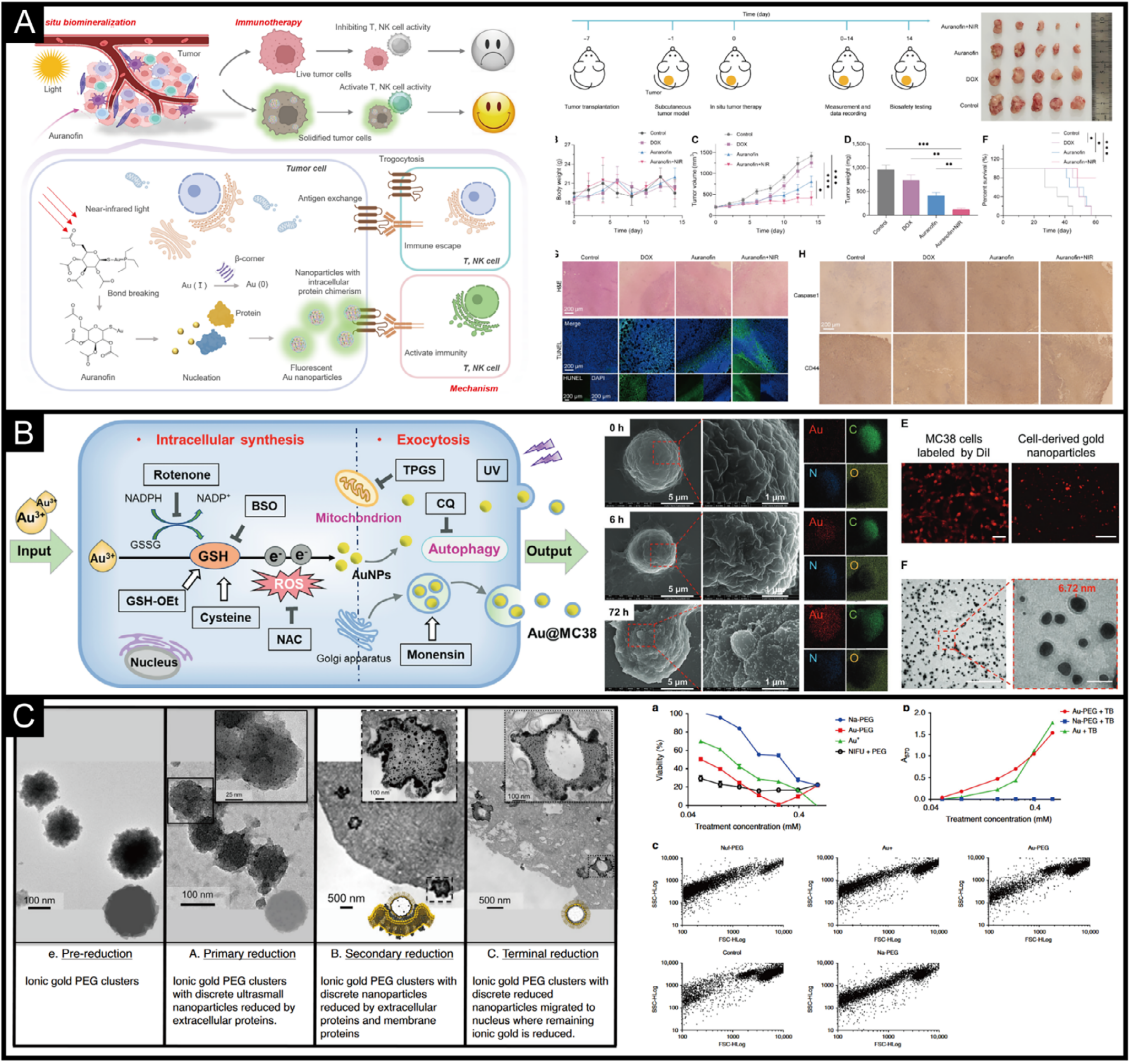
(A) The schematic diagram and anti‐tumor effect of gold complex anticancer drugs forming gold IPHMs within tumor cells to form a tumor whole cell vaccine [215]. Copyright 2024, American Association for the Advancement of Science. (B) The mechanism of intracellular synthesis and extracellular secretion of gold IPHMs, as well as the morphological characterization of cells and IPHMs [245]. Copyright 2021, Wiley‐VCH. (C) The formation process of PEG‐Au‐based intracellular gold IPHMs and the therapeutic effect on tumors [224]. Copyright 2020, Springer Nature.

The inorganic structure of IPHMs has excellent physical and chemical properties, and the surface protein structure provides a basis for biosafety. And it has unique characteristics and biological functions, which can produce or remove active substances, maintain redox homeostasis, and promote disease treatment [206]. The physical and chemical properties that can be applied to biomedical treatment include photothermal effects, photodynamic effects, catalytic effects, immune effects, and so on [207, 208, 209, 210, 211]. At the same time, the presence of proteins can make IPHMs easily metabolized in the body by the liver, kidneys, and skin outside the body [21, 212, 213]. Metal drugs are induced to biomineralize and produce IPHMs by proteins with specific structures in the body [214, 215]. Auranofin immobilizes tumor cells by biomineralization in tumor cells to produce gold nanoclusters, leading to pyroptosis (Figure 5A). Under near‐infrared light irradiation, this phenomenon can be promoted, and immunotherapy that suppresses cell trogocytosis can be achieved through biomineralized IPHMs [215]. Platinum‐based drugs have also been found to produce platinum nanoparticles in the bloodstream [180]. The surface of tumor cells is rich in extracellular matrix, which is the structural protein basis that can promote the production of IPHMs [216, 217]. Tumor cell death can be caused by stimulating surface calcification or silicification, and calcified or silicified tumor cells are similar to tumor whole cell vaccines, which can achieve immune suppression of tumor proliferation [218, 219]. This type of silicification or calcification is not only used for tumor treatment but also on the surface of red blood cells, which can shield red blood cell surface antigens while maintaining their oxygen transport function.

The application prospects of in vitro synthesis of IPHMs based on phase transition proteins are also extensive. Metal nanoparticles based on BSA exhibit different properties [100, 220]. Iron and platinum based IPHMs have catalytic properties and photodynamic effects, which are needed in the treatment of diseases that require reactive oxygen species [221, 222, 223]. Gold, silver, and copper based IPHMs have photothermal and photodynamic effects and play a role in the treatment of inflammation or tumors [224, 225, 226]. The different substances and structures produced by different ions result in different properties. The screening of phase transition proteins can be based on the properties of inorganic ions and the required functions. For example, gold based IPHMs may exhibit fluorescence effects when they are small in size, photothermal effects, and plasmon resonance effects when they are at the nanoscale and larger surface areas when they are at the micrometer level [52]. Some IPHMs have unstable structures, and inorganic ions and proteins are released into the microenvironment. This responsive design can also be applied [148, 227, 228]. In addition, the surface of IPHMs generated within cells will be embedded with protein or membrane structures (Figure 5B). The properties and sizes of the formed IPHMs are relatively uniform, and the mode of protein chimerism in inorganic structures may not yield responsive results.

The crystalline and amorphous states of inorganic structures in IPHMs can affect their properties. The catalytic effect of bacteria producing amorphous IPHMs is relatively strong, and they can be used as in situ synthesized nanoenzymes for the treatment of intestinal inflammation [229]. Or copper ions can be embedded in protein aggregates in tumor cells to form single atom nanoenzymes to kill tumor cells [51]. This method of in situ generation of IPHMs poses new requirements for the delivery of inorganic ions. Polyethylene glycol is used to transport gold ions for enrichment and biomineralization at tumor sites to produce IPHMs [230, 231]. When polyethylene glycol comes into contact with tumor cell membranes, it releases gold ions, induces heavy metal ion poisoning in tumor cells, and can inhibit tumor tissue proliferation (Figure 5C). The different delivery methods will affect the properties of the generated IPHMs [232, 233, 234]. During the process of blood movement, the protein structure on the surface of IPHMs changes, which means that proteins in the blood exchange with surface proteins, leading to changes in the properties of IPHMs [212, 213, 235]. Another phenomenon is that proteins in the blood adsorb another layer of protein coronas on the surface of IPHMs.

The morphology of IPHMs in the body is sometimes a dynamic process due to the complex microenvironment within the body [4, 236, 237]. Calcium based IPHMs will further crystallize and grow in the enamel or bone structure, embedding into bone tissue. This approach is suitable for research on tissue engineering repair [238, 239]. Additionally, it may also aggravate the pathological characteristics of atherosclerosis or hepatolithiasis, which is a possible side effect [240, 241]. When IPHMs are metabolized by the kidneys, aggregates are formed in the lysosomes of IPHMs glomerular cells. Eventually metabolized out of the body [242, 243, 244]. Therefore, while studying the therapeutic role of IPHMs, the concentration, morphology, size, performance, and structural stability of IPHMs need to be considered. There are dynamic changes in the structure of IPHMs in different microenvironments in the body, which can affect the physical and chemical properties, metabolic modes, and pathways of IPHMs.

### 4.2 Disease Diagnostic Imaging Based on IPHMs

**FIGURE 6:**
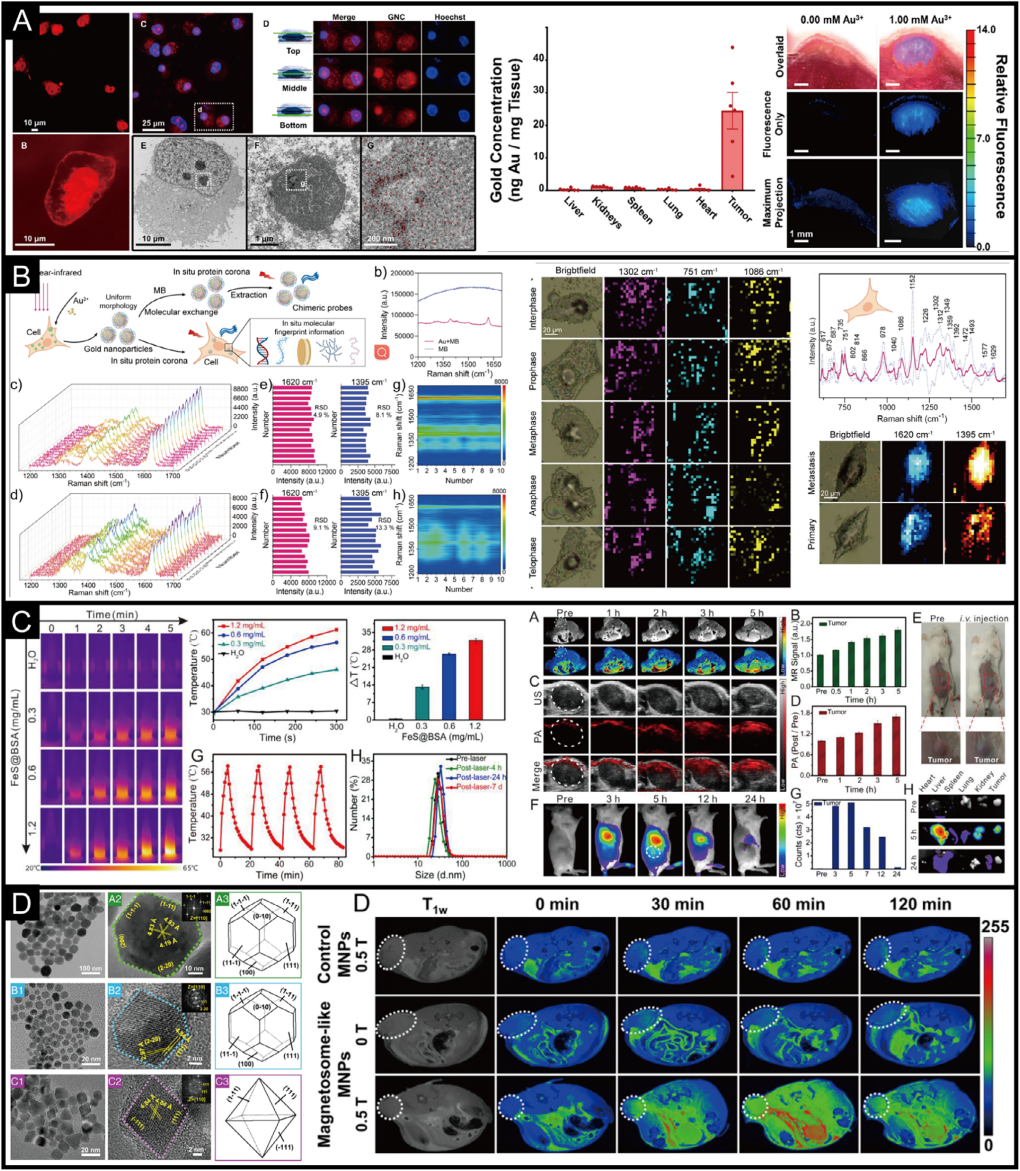
(A) Fluorescence IPHMs for imaging of cells and tumor tissues [230]. Copyright 2024, American Chemical Society. (B) The surface enhanced Raman scattering (SERS) activity and stability of IPHMs, as well as the SERS mapping of tumor cells [53]. Copyright 2024, Wiley‐VCH. (C) The photothermal effect of IPHMs and their thermal related imaging. Such as photoacoustic imaging (PAI), thermal imaging, etc. [221]. Copyright 2020, Elsevier. (D) The nanostructure of hybrid magnetosomes and their magnetic resonance imaging (MRI) of tumor tissues [71]. Copyright 2022, U.S. National Academy of Sciences.

The optical properties of IPHMs are widely used in disease diagnosis because they can visualize diseased tissues or inflammatory sites. Metal nanoclusters or fluorescent rare earth quantum dots synthesized by proteins can be used for fluorescence imaging of tumor tissues and cells [9, 246, 247]. The doping of rare earth elements can lead to the emission of longer wavelength near‐infrared fluorescence from fluorescent quantum dots or nanoclusters [248, 249, 250]. The synthesis conditions can be optimized to make the fluorescent IPHMs have longer Stokes shifts and narrower half‐peak widths, which is favorable for multifluorescence imaging and avoids fluorescence overlap. The difference in serum concentration in cell culture medium can affect the fluorescence effect of fluorescent gold nanoparticles [230]. Unlike phosphate buffer saline (PBS) as a culture buffer, fluorescent gold nanoparticles are produced in the nucleus (Figure 6A). Fluorescence imaging can be achieved on tumor tissue while it is enriched. Not only within cells, bacteria can form a biomineralized microenvironment through phase separation, leading to the synthesis of rare earth quantum dots with emission wavelengths up to near‐infrared [50, 144, 251]. The outer shell protein on the surface of the virus produces monodisperse fluorescent quantum dots, which can be used to monitor the process of virus infection in cells [203].

There are not many types of IPHMs with surface plasmon resonance (SPR) effects, mainly concentrated on specific sized nanoparticles formed by precious metal elements, such as gold, silver, and copper [252, 253, 254]. Gold nanoparticles mineralized within cells exhibit SERS activity, which can provide molecular fingerprint information of in situ protein coronas [52, 53, 255]. The IPHMs can recognize Raman shifts at 617, 751, 1040, 1302, and 1629 cm^−1^, which belong to C─C twisting (protein), DNA, collagen, Amide III (β sheet structure), and Amide I (Amide C═O stretching absorption for the β‐form polypeptide films) [256, 257, 258, 259]. The stable structure and uniform size of this IPHMs result in high stability of their SERS signals (Figure 6B). At the same time, IPHMs synthesized in situ within cells do not alter in situ molecular information, unlike the surface protein molecules of IPHMs introduced from the outside that are not in situ [53]. This advantage can be used to obtain in situ SERS mapping of cells to analyze the process of tumor cell proliferation and division. In addition, IPHMs synthesized by phase transition proteins can also be used for SERS analysis, with the advantage of being able to see tag molecules embedded in the protein to form stable SERS signals [52]. The chiral synthesis of IPHMs from amino acids can also affect SERS hotspots, which should also be taken into consideration when designing IPHMs with SERS activity.

The photothermal effect of IPHMs will also be applied in disease imaging and diagnosis. Photoacoustic imaging is achieved using the photothermal effect of IPHMs [260, 261]. When IPHMs absorb the energy of photons and then release heat in a non radiative transition, nearby molecular vibrations and thermal expansion generate acoustic signals [7, 262]. The higher the efficiency of converting light energy into heat, the more obvious the imaging effect. Under near‐infrared light irradiation, the temperature of IPHMs synthesized by phase transition proteins can rise to 57°C (Figure 6C). While obtaining photoacoustic imaging results of mouse tumor tissue, the thermal signals generated by photothermal effects can also be used for photothermal imaging [221]. Not only photoacoustic imaging, but photothermal optical coherence tomography is also a method of imaging molecular vibrations caused by photothermal effects [6, 262–264]. However, it should be noted that the depth of light penetration is a factor that affects the diagnostic imaging effect.

Magnetic IPHMs can be applied in nuclear magnetic imaging, such as iron, gadolinium, etc. [72, 265–267]. This type of metal element is relatively active and can be synthesized by phase transition proteins, as well as by bacteria or cells [268, 269]. The ions chelated on the protein also have the effect of nuclear magnetic imaging [71]. The magnetism generated by different forms of magnetosomes varies, but they can all achieve magnetic resonance imaging of tumors (Figure 6D). Therefore, when designing such IPHMs, the selection of inorganic elements is the most crucial.

## 5 Summary and Outlook

In summary, this article reviews the design ideas and principles of IPHMs applied in the biomedical field. From the perspective of proteins and cells, integrate the perspectives and methods of synthetic biology and materials science in the synthesis of IPHMs. This includes two synthetic approaches and methods: in vitro synthesis of IPHMs and in situ biomineralization of IPHMs within cells. Then, the relevant directions and design ideas of diagnostic imaging and treatment of IPHMs in biomedical fields were summarized and analyzed, including photothermal therapy, photodynamic therapy, catalytic therapy, photoacoustic imaging, magnetic resonance imaging, and fluorescence imaging, among others. Importantly, the design principles of IPHMs have been proposed to provide a research foundation for scholars in the fields of synthetic biology and materials science for future research.

Secondly, the components of IPHMs are proteins and inorganic structures. This endows IPHMs with the biological activity of proteins and the physicochemical properties of inorganic materials, making them a versatile hybrid material with a wide range of applications. In subsequent research, proteins or peptides with specific biological functions can be designed or screened, such as targeting, anti‐tumor, responsiveness, etc. And it can be combined with the characteristics of inorganic nanostructures, such as photothermal effect, magnetism, fluorescence, etc. This can achieve cascading or synergistic disease diagnosis and treatment. The reactor for the biosynthesis of IPHMs is the active unit, which includes cells, bacteria, etc. The functionality and biological activity of this active unit are more abundant. Exploring the role of composite structures of active units and IPHMs in biomedical applications is an interesting question. The tumor cells that produce IPHMs may achieve anti‐tumor immunotherapy similar to tumor vaccines, and the physical and chemical properties of IPHMs can be utilized to enhance efficacy. This is not just a disease like cancer, other diseases such as sepsis, Alzheimer's, and poor sperm development can also be considered for treatment with IPHMs. More application directions need to be further explored.

## Conflicts of Interest

The authors declare no conflicts of interest.
